# Obesity-Associated Metabolic Disturbances Reverse the Antioxidant and Anti-Inflammatory Properties of High-Density Lipoproteins in Microglial Cells

**DOI:** 10.3390/biomedicines9111722

**Published:** 2021-11-19

**Authors:** Elena Grao-Cruces, Maria C. Millan-Linares, Maria E. Martin-Rubio, Rocio Toscano, Sergio Barrientos-Trigo, Beatriz Bermudez, Sergio Montserrat-de la Paz

**Affiliations:** 1 Department of Medical Biochemistry, Molecular Biology, and Immunology, School of Medicine, University of Seville, 41009 Seville, Spain; egrao@us.es (E.G.-C.); mtoscanos@us.es (R.T.); 2 Department of Food & Health, Institute of Fat, Spanish National Research Council, 41013 Seville, Spain; mcmillan@ig.csic.es; 3 Department of Cell Biology, Faculty of Biology, University of Seville, 41012 Seville, Spain; mariamartin@us.es (M.E.M.-R.); bbermudez@us.es (B.B.); 4 Department of Nursing, Faculty of Nursing, Physiotherapy and Podiatry, University of Seville, 41009 Seville, Spain; sbarrientos@us.es

**Keywords:** obesity, paraoxonase, lipoproteins, oxidation, microglia, neuroinflammation

## Abstract

High-density lipoproteins (HDLs) play an important role in reverse cholesterol transport and present antioxidant properties, among others. In the central nervous system (CNS), there are HDLs, where these lipoproteins could influence brain health. Owing to the new evidence of HDL functionality remodeling in obese patients, and the fact that obesity-associated metabolic disturbances is pro-inflammatory and pro-oxidant, the aim of this study was to investigate if HDL functions are depleted in obese patients and obesity-associated microenvironment. HDLs were isolated from normal-weight healthy (nwHDL) and obese men (obHDL). The oxHDL level was measured by malondialdehyde and 4-hydroxynoneal peroxided products. BV2 microglial cells were exposed to different concentrations of nwHDL and obHDL in different obesity-associated pro-inflammatory microenvironments. Our results showed that hyperleptinemia increased oxHDL levels. In addition, nwHDLs reduced pro-inflammatory cytokines’ release and M1 marker gene expression in BV2 microglial cells. Nevertheless, both nwHDL co-administered with LPS+leptin and obHDL promoted BV2 microglial activation and a higher pro-inflammatory cytokine production, thus confirming that obesity-associated metabolic disturbances reverse the antioxidant and anti-inflammatory properties of HDLs in microglial cells.

## 1. Introduction

High-density lipoproteins (HDLs) are circulating particles; beyond serving as lipid transporters, they carry out a wide number of biological functions [1]. The fundamental activity of HDLs is cholesterol transport from peripheral tissues to the liver for catabolism and excretion into the bile. Moreover, in physiological conditions, HDLs have been recently reported to exert other functions, including anti-inflammatory and antioxidant capacity, highly dependent on HDL composition [2]. The antioxidant capacity of HDLs is mainly conferred by the presence of apolipoproteins and enzymes transported by HDLs, including paraoxonase 1 (PON1) [3]. In addition, lipid components are essential in HDL-related antioxidant capacity in order to prevent their own oxidation and low-density lipoproteins (LDLs) oxidation [4]. It should be noted that oxidative modifications of HDLs participate in the impairment of HDL functionality [5]. Indeed, the oxidation of HDLs occurs in different pathological conditions characterized by an oxidant microenvironment increased, such as obesity, diabetes, inflammatory, or renal diseases [6,7,8,9]. HDLs isolated from patients with pathological conditions were shown to have a reduced antioxidant capacity [10,11,12].

Obesity is known to be associated with circulating hyperleptinemia. Leptin is a circulating hormone with cytokine-like actions mainly produced by adipose tissue. In epidemiological studies, a direct correlation of plasma leptin levels with body fat mass has been described [13]. The most prominent effect of leptin is appetite control, but it has become increasingly clear that leptin also influences the immune system [14]. As part of its immune-modulating actions, leptin is able to increase oxidative stress and activation of monocytes [15] and T cells [16] and mediates homeostasis in a variety of immune cells [17]. In the central nervous system (CNS), leptin activates ObRb in microglial cells and induces interleukin (IL)-1β, IL-6, and tumor necrosis factor (TNF)-α production [18].

Microglia-mediated neuroinflammation is considered to play an important role in the pathogenesis and progression of neurodegenerative diseases [19]. Similar to periphery macrophages, it is well-known that microglia can alter their phenotypes and functions in response to microenvironmental disturbances. Therefore, different stimuli, such as lipopolysaccharide (LPS), are used to model neuroinflammation associated with neurodegeneration. By acting at its receptors, LPS activates various intracellular molecules, which alter the expression of a plethora of inflammatory mediators [20]. Depending on the predominance of secreted factors, microglia have been characterized to express the classical activation phenotype (M1, pro-inflammatory) or the alternative activation phenotype (M2, anti-inflammatory) [21]. The M1 state causes the release of proinflammatory and pro-oxidant mediators with increased expression of CD80 and iNOS. The M2 state is associated with the expression of the anti-inflammatory and antioxidant mediators, as well as CD200R and arginase-1 (Arg1) [22,23]. Alterations in microglia M1/M2 polarization have been associated with neurodegenerative diseases [24,25]. Neuropathologies triggered by metabolic syndrome, which includes diabetes and obesity, often result from increased permeability of the blood–brain barrier (BBB), rising the entry of toxins, immune cells, pathogens, and molecules, as lipoproteins, into the brain [26]. In obesity, HDLs can cross the BBB and perform the same functions as in other tissues, reverse cholesterol transport, and antioxidant and anti-inflammatory activities.

The consequence of oxidation on the protective role of HDL against the leptin-induced oxidation on microglial cells remain unknown. Taken together, the aim of this study was to investigate the effects of leptin-induced oxidation on HDL isolated from healthy subjects and HDL isolated from obese individuals with hyperleptinemia on microglial cells.

## 2. Materials and Methods

### 2.1. Subjects and Ethics

This study was conducted according to Good Clinical Practice Guidelines and in line with the principles outlined in the Helsinki Declaration of the World Medical Association. Ethics approval was obtained from the Human Clinical Research and Ethics Committee of the University Hospital Virgen Macarena (PI00082017), and all subjects gave written informed consent. This study included 20 normal-weight (body mass index (BMI) 18–24.9 kg/m^2^ and leptin levels less than 7 ng/mL) and 20 obese (BMI > 30 kg/m^2^ and leptin levels over than 40 ng/mL) subjects. Inclusion criteria included age from 18 to 45 years and no known diseases or taking medication for dyslipidemia, diabetes, hypertension, or any other metabolic disorders. Males were only included in this study, given the known differences in lipoproteins by sex and timing of menstrual cycle [27].

### 2.2. Clinical Measurements

Subjects’ weight, height, waist and neck circumference, and body-fat percentage were measured by trained recruiters. Weight (nearest 0.1 kg), height (nearest 0.1 cm), and body-fat percentage were measured by using a TANITA Body Composition Analyzer (ModelBC-545N). Waist circumference was measured exactly midway between the lowest rib and the peak of the iliac crest. Neck circumference was measured at the midway point of the neck to 0.5 cm just below the laryngeal prominence. Measurements of systolic and diastolic blood pressure (SBP and DBP) were performed with a standard method. BMI was calculated as weight (kg)/height (m)^2^. Lifestyle factors (physical activity, alcohol consumption, and cigarette smoking) were obtained by standardized questionnaires.

### 2.3. Biochemical Measurements

Fasting venous blood samples were taken at 8:00 after a 12 h overnight fast. Glucose was immediately measured by using the glucose/oxidase method (Glucose GOD-PAP; Biolabo, Madrid, Spain). Insulin was measured by using enzyme-linked immunosorbent assay (Diagnostic System Laboratories, Webster, TX, USA). Total cholesterol and triglycerides were determined by enzymatic methods (CHOD-PAP and GPO-PAP, respectively; Roche Diagnostics, Basel, Switzerland). HDL cholesterol was determined after precipitation with phosphotungstic acid. LDL cholesterol was measured by using an Advia 2400 Clinical Chemistry System (Siemens Healthcare Diagnostics, Erlangen, Germany). NEFAs were measured with an ACS-ACOD assay (Wako Chemicals GmbH, Neuss, Germany). Glycated hemoglobin (HbA1c) was measured according to the Standard Operating Procedure of the IFCC Reference, with an automated high-performance liquid chromatographic analyzer (Bio-Rad, Milan, Italy).

### 2.4. HDL Isolation and Oxidized HDL Measurement

For HDL isolation, blood was collected into K_2_EDTA (Becton-Dickinson, San Jose, CA, USA) tubes, centrifugated (140× *g*, 10 min at 4 °C), and the plasma removed and stored at −80 °C until analyzed. The HDL fraction was isolated from 200 µL of plasma by density gradient ultracentrifugation (Beckman Optima TLX, TLA 100.2 rotor, Indianapolis, IN, USA). Sodium bromide (NaBr, Sigma-Aldrich, Madrid, Spain) and potassium bromide (KBr, Sigma-Aldrich) salt solutions were used to adjust the density of the plasma as follows: 200 μL plasma was mixed with 1.182 g/mL NaBr to increase the density to 1.019 g/mL. The plasma was then overlaid with KBr salt solution (density 1.019 g/mL) to a final volume of 1.0 mL and centrifuged (100,000 rpm, 16 °C, 2 h). The VLDL (combined with IDL) fraction (400 μL) was aspirated from the top of the centrifuge tubes. Then, the remaining mixture was adjusted to a density of 1.063 g/mL, with NaBr and overlaid to 1.0 mL with 1.182 g/mL KBr solution. LDL was isolated by centrifugation (100,000 rpm, 16 °C, 3 h) and aspiration of the top layer (400 μL). The infranatant was adjusted to a density of 1.21 g/mL by the addition of 1.488 g/mL NaBr, and overlaid to 1.0 mL with 1.21 g/mL KBr solution and centrifuged (100,000 rpm, 16 °C, 15 h). The HDL fraction (400 μL) was then aspirated carefully from the top of each tube. Prior to HDL functional assessment, the isolated HDL fractions were dialyzed against phosphate-buffered saline (PBS), in the dark, at 4 °C, with three buffer changes over 24 h to remove salts. The amount of total protein was assayed in the isolated HDL fraction according to manufacturer′s kit instructions, using the bicinchoninic acid assay (BCA, Pierce Biotechnology BCA™ Protein Assay Kit, Thermo Scientific, Waltham, MA, USA). Briefly, the BSA standards and samples (25 μL) were mixed with working reagent (200 μL) and aliquoted into 96-well plates. The plates were then incubated at 37 °C for 30 min, and the absorbance (at 562 nm) measured. The protein concentration of each sample was calculated by comparison to a standard calibration curve (0–1 mg/mL BCA). To evaluate oxidized HDL levels in plasma, Oxiselect Human Oxidized HDL ELISA Kit (Cell Biolabs, San Diego, CA, USA), an enzyme immunoassay containing 4-hydroxynonenal-(HNE-)HDL standard with a detection sensitivity limit of 2 ng/mL, was used.

### 2.5. Paraoxonase-1 Levels and Activity Assay

Serum samples were diluted 1:10 in phosphate buffer containing 2 mmol/L CaCl_2_ (pH 8). Diluted serum was added to 96-well plates in triplicate, and paraoxon-ethyl substrate (Sigma, D9286, Madrid, Spain) was added. Absorbance at 405 nm was measured at 30 s intervals over 20 min. Paraoxonase-1 activity was expressed as IU/L serum. ELISA kit (ThermoFisher Scientific, Madrid, Spain) was used to measure serum concentrations of paraoxonase-1 and results were expressed as ng/mL.

### 2.6. BV2 Microglial Cell Culture and Treatments

The immortalized murine microglial cell line BV2 was purchased from the Instituto de la Grasa (Seville, Spain). Cells were routinely cultured in high-glucose Dulbecco′s modified Eagle′s medium (DMEM) supplemented with 10% heat-inactivated fetal bovine serum (FBS) and 1% penicillin/streptomycin (P/S). Culture conditions were 5% CO_2_ at 37 °C. Subcultures were made every 3 days, using 0.25% trypsin-EDTA. To maintain cells in an undifferentiated state, they were passaged before obtaining confluence. Only BV2 microglial cells at culture passages 5 to 10 were used for the experiments. Before the treatment, BV2 microglial cells were seeded at a density of 5 × 10^5^ cells/mL in 12-well plates for 24 h in high-glucose DMEM supplemented with 1% heat-inactivated FBS and 1% P/S. BV2 microglia cells were exposed to LPS (100 ng/mL) for additional 3 h. To assess the effect of HDL on microglia polarization, BV2 microglial cells were exposed to HDL isolated from normal-weight men (nwHDL) and HDL isolated from obese men (obHDL) at 250, 500, and 800 μg/mL during 24 h. Recombinant human Leptin (PeproTech, London, UK) was added at 10 ng/mL to the medium prior to 30 min incubation.

### 2.7. RNA Isolation and RT-qPCR Analysis

Total RNA was extracted by using TRIsure Reagent (Bioline, London, UK), as instructed by the manufacturer. A_260_/A_280_ ratio in a NanoDrop ND-1000 Spectrophotometer (Thermo Scientific) was used to determinate RNA quality. Momentarily, RNA (1 µg) was subjected to reverse transcription (iScript, Bio-Rad, Madrid, Spain). An amount of 10 ng of the resulting cDNA was used as a template for real-time PCR amplifications. The mRNA levels for specific genes were determined in a CFX96 system (Bio-Rad). For each PCR reaction, cDNA template was added to Brilliant SYBR green QPCR Supermix (Bio-Rad) containing the primer pairs for either gene or for glyceraldehyde 3-phosphate dehydrogenase (GAPDH) and hypoxanthine phosphoribosyltransferase 1 (HPRT) as housekeeping genes (Supplementary Materials Table S1). All amplification reactions were performed in triplicate, and average threshold cycle (Ct) numbers of the triplicates were used to calculate the relative mRNA expression of candidate genes. The magnitude of change of mRNA expression for candidate genes was calculated by using the standard 2^−(ΔΔCt)^ method. All data were normalized to endogenous reference (GAPDH and HPRT) gene content and expressed as percentage of controls.

### 2.8. Statistical Analysis

All values are expressed as arithmetic means ± standard deviations (SD). Data were evaluated with Graph Pad Prism Version 6.01 software (San Diego, CA, USA). The statistical significance of any difference in each parameter among the groups was evaluated by one-way analysis of variance (ANOVA), following a Tukey multiple comparisons test as post hoc test. Pearson’s *r*-value was used to analyze the statistical significance of correlation test. The *p*-values less than 0.05 were considered to be statistically significant.

## 3. Results

### 3.1. Clinical and Biochemical Characteristics of the Study Subjects

Demographic characteristics and clinical and biochemical measurements of the study participants are presented in Table 1. Participants with obesity had significantly higher BMI, waist and neck circumferences, and percent body fat relative to normal-weight participants (all *p* < 0.001). While other variables, such as systolic and diastolic blood pressure, NEFAs, total triglycerides and cholesterol, LDL-C, glucose, and HbA1c, appeared to be higher in participants with obesity than those with a normal weight, these differences were not statistically significant. Meanwhile, HDL-C was slightly lower in participants with obesity compared to those with a normal weight (*p* = 0.111).

### 3.2. Serum Leptin Level Is Positively Correlated with Oxidized HDL Levels

**Figure 1:**
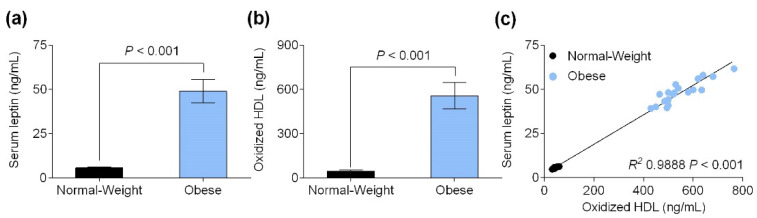
(**a**) Serum leptin levels (ng/mL) and (**b**) oxidized HDL content (ng/mL) in normal-weight volunteers and obese subjects. (**c**) Correlation between serum leptin levels and oxidized HDL content. Values are presented as means ± SD (*n* = 20).

As shown in Figure 1a, all the obese individuals met the criteria for hyperleptinemia (over than 40 ng/mL), and compared to participants with a normal weight, their serum leptin levels were significantly higher (48.97 ± 1.49 ng/mL vs. 5.742 ± 0.09 ng/mL, *p* < 0.001). Additionally, participants with obesity showed higher oxidized HDL levels than those participants with a normal weight (557.0 ± 19.94 ng/mL vs. 45.35 ± 1.61 ng/mL, *p* < 0.001, Figure 1b). As depicted in Figure 1c, the serum leptin level was significantly positively correlated with the oxidized HDL level (*r*^2^ 0.9888, *p* < 0.001).

### 3.3. Obesity-Associated Hyperleptinemia Reduced Paraoxonase-1 Levels and Activity

**Figure 2:**
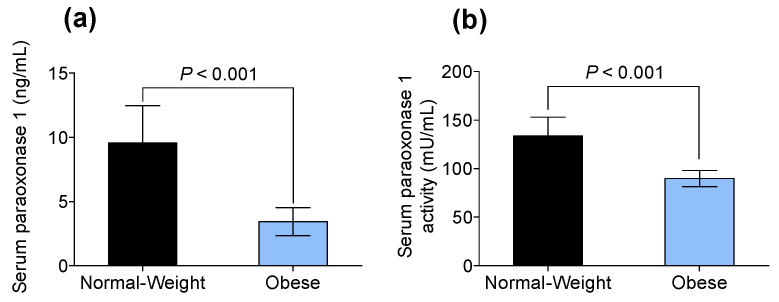
Serum paraoxonase-1 (**a**) levels (ng/mL) and (**b**) activity (mU/mL) in normal-weight volunteers and obese subjects. Values are presented as means ± SD (*n* = 20).

The impact of variations in paraoxonase-1 levels and activity on obese individuals compared to participants with normal weight is shown in Figure 2. There was a significant decrease in the serum paraoxonase-1 levels in individuals with obesity-associated hyperleptinemia and compared to those participants with a normal weight (3.44 ± 1.08 ng/mL vs. 9.57 ± 2.89 ng/mL, *p* < 0.001, Figure 2a). Similarly, participants with obesity showed lower serum paraoxonase activity than those participants with a normal weight (89.80 ± 8.25 mU/mL vs. 133.73 ± 19.37 mU/mL, *p* < 0.001, Figure 2b). In order to explore whether differences in paraoxonase activity were due to leptin levels, we used Pearson’s *r*-value. Serum paraoxonase activity correlated negatively with leptin levels in both normal-weight and obese individuals (*r*^2^ 0.9434, *p* < 0.001).

### 3.4. HDL Isolated from Hyperleptinemic Obese Subjects Increased Pro-Inflammatory Cytokine Expression in Microglial Cells

**Figure 3:**
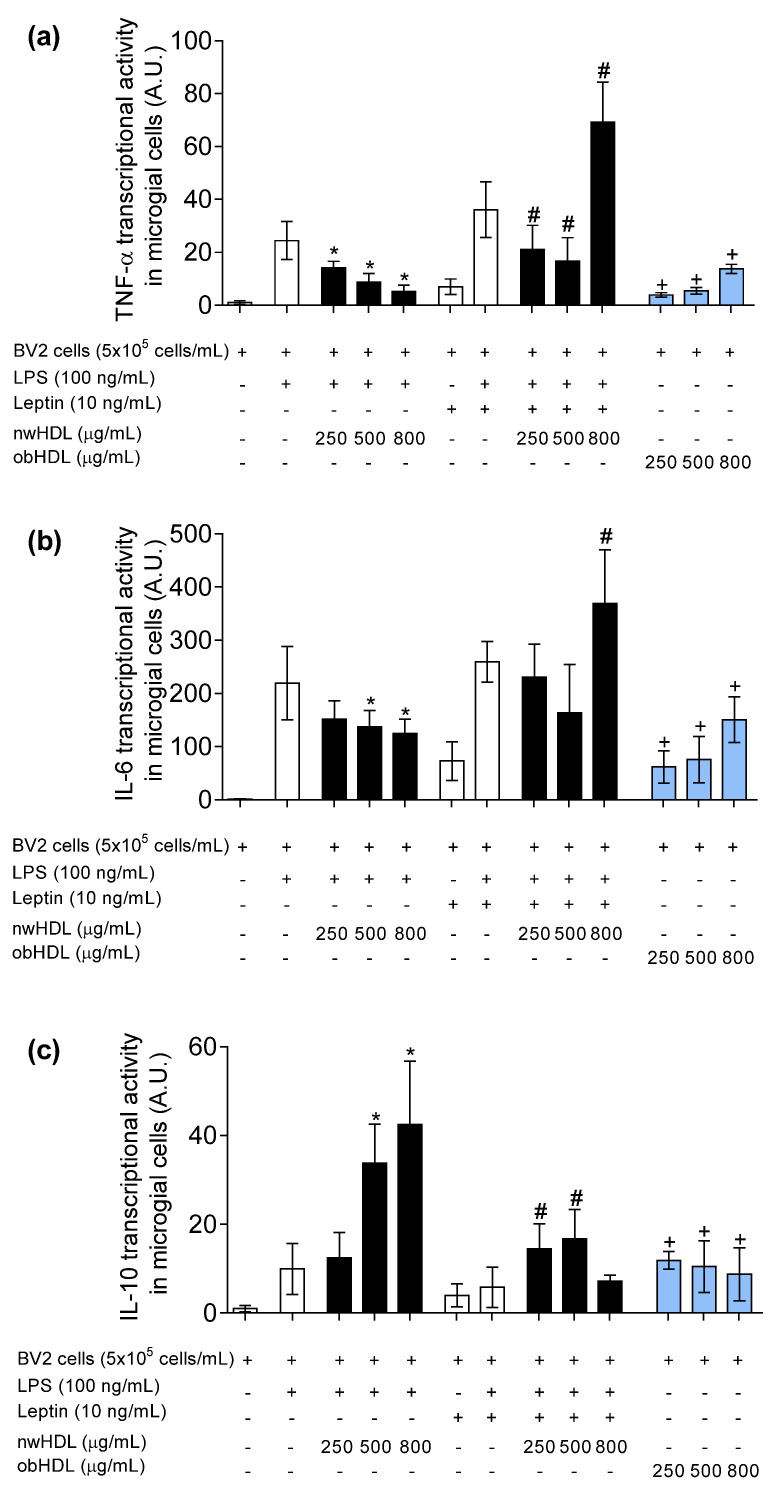
Transcriptional activity (A.U.) of (**a**) TNF-α, (**b**) IL-6, and (**c**) IL-10 genes. BV2 microglial cells were incubated with LPS (100 ng/mL) and leptin (10 ng/mL) and treated with HDL isolated from normal-weight volunteers (nwHDL) or from obese subjects (obHDL) at 250, 500, and 800 μg/mL during 24 h. Values are presented as means ± SD (*n* = 6). * *p <* 0.05 vs. LPS, # *p* < 0.05 vs. LPS + leptin, + *p* vs. untreated cells.

To further explore the possible role of obesity-associated hyperleptinemia on HDL functionality, we analyzed cytokine expression in microglial cells. As shown in Figure 3, LPS treatment increased the expression of pro-inflammatory cytokines, such as TNF-α (Figure 3a) and IL-1β (Figure 3b). All the doses of normal-weight HDL (nwHDL) had a significantly anti-inflammatory effect in LPS-stimulated BV2 microglial cells. The addition of leptin into culture media enhanced the pro-inflammatory effect of LPS and reverted the anti-inflammatory capacity of normal-weight HDL at the higher dose (800 µg/mL) skewing to pro-inflammatory dysfunctional HDL particle. In line with these results, in absence of LPS and leptin into the culture media, the treatment with HDL isolated from obese (obHDL) subjects had significantly pro-inflammatory effects in gene expression of TNF-α and IL-1β at all the doses assayed in BV2 microglial cells. Regarding anti-inflammatory IL-10 gene expression (Figure 3c), the mRNA levels of IL-10 were overrepresented by nwHDL treatment in the presence of LPS and leptin. However, the capacity to increase IL-10 mRNA levels was diminished with obHDL treatment.

### 3.5. HDL Isolated from Hyperleptinemic Obese Subjects Promotes M1 Polarization in Microglial Cells

**Figure 4:**
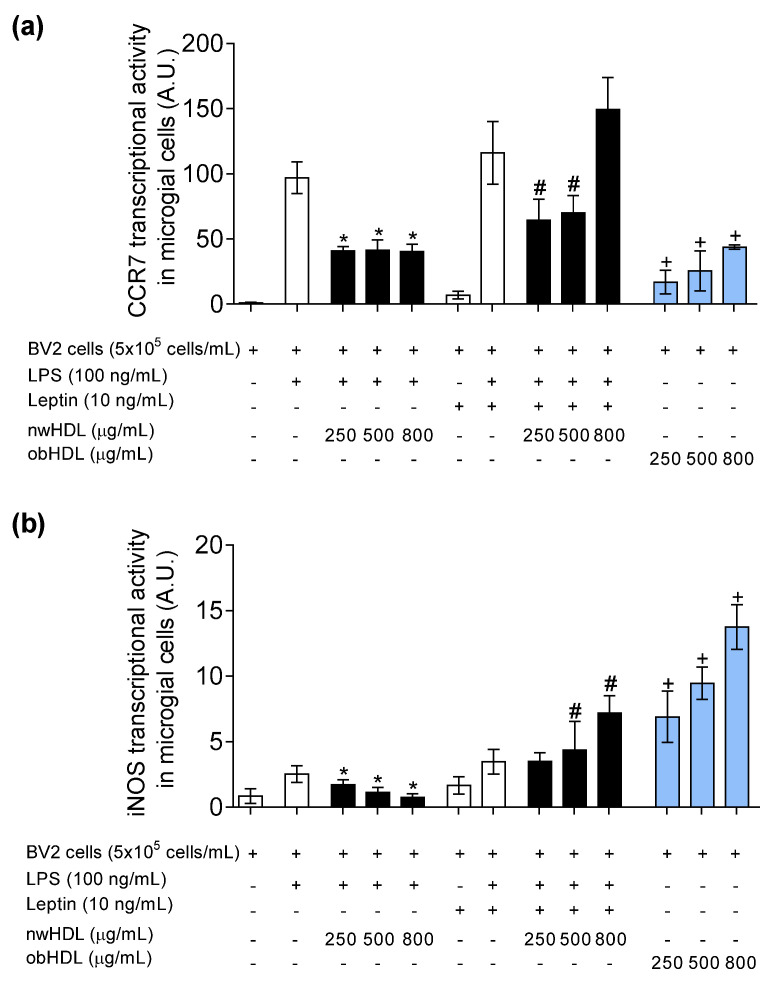
Transcriptional activity (A.U.) of (**a**) CCR7 and (**b**) iNOS M1 marker genes. BV2 microglial cells were incubated with LPS (100 ng/mL) and leptin (10 ng/mL) and treated with HDL isolated from normal-weight volunteers (nwHDL) or from obese subjects (obHDL) at 250, 500, and 800 μg/mL during 24 h. Values are presented as means ± SD (*n* = 6). * *p <* 0.05 vs. LPS, # *p* < 0.05 vs. LPS + leptin, + *p* vs. untreated cells.

**Figure 5:**
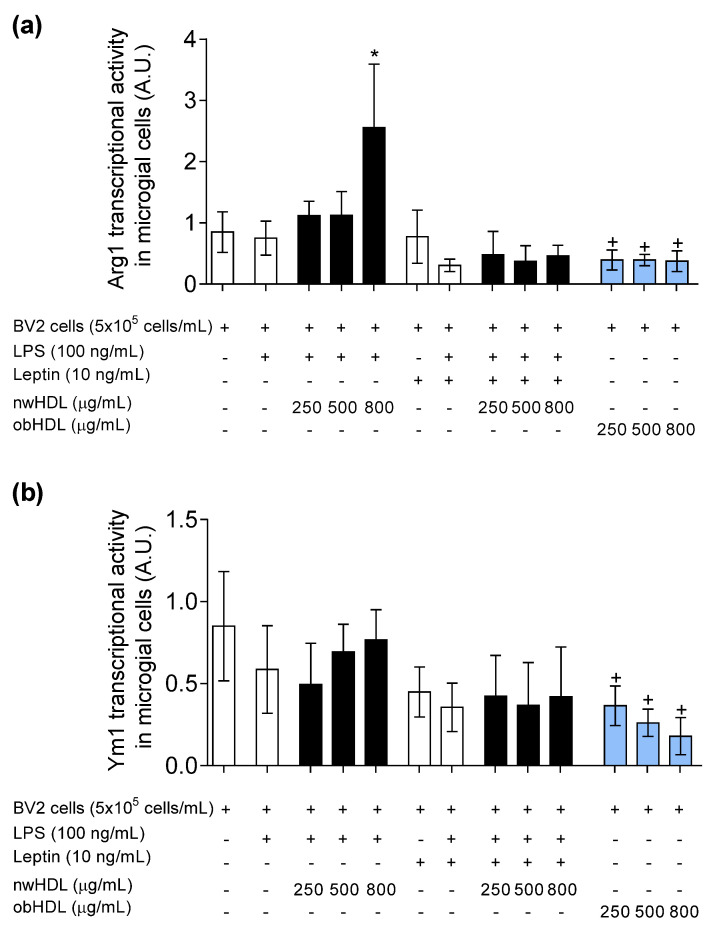
Transcriptional activity (A.U.) of (**a**) Arg1 and (**b**) YM1 M2 marker genes. BV2 microglial cells were incubated with LPS (100 ng/mL) and leptin (10 ng/mL) and treated with HDL isolated from normal-weight volunteers (nwHDL) or from obese subjects (obHDL) at 250, 500, and 800 μg/mL during 24 h. Values are presented as means ± SD (*n* = 6). * *p <* 0.05 vs. LPS, + *p* vs. untreated cells.

In gaining deeper insight into the role of HDL on microglia polarization under the influence of LPS and leptin, we observed that nwHDL downregulated the transcriptional activity of M1 gene markers, CCR7 (Figure 4a) and iNOS (Figure 4b). In contrast, obHDL not only promoted the mRNA levels of CCR7 and iNOS but also promoted the transcriptional activity in absence of leptin and LPS. Furthermore, the gene expression of anti-inflammatory M2 phenotypic markers Arg1 (Figure 5a) and Ym1 (Figure 5b) was analyzed. Moreover, nwHDL upregulated the Arg1 gene expression at 800 µg/mL; however, at the same dose, in the presence of leptin, nwHDL lost its M2-promoting capacity. In contrast, at all doses assayed, obHDL downregulated the mRNA levels of Arg1 and Ym1 in BV2 microglial cells.

## 4. Discussion

Neuroinflammation is the onset of several neurodegenerative diseases. Microglial cells develop a key role in the management of inflammation; microglia differentiate into a wide range of phenotypes, and the main ones are M1 and M2 phenotypes, pro- and anti-inflammatory, respectively [19,20]. Inflammation is an essential process to maintain physiological status; the M1 phenotype is in charge of the immune response, and the anti-inflammatory phenotype is crucial to recover the tissue and maintain the homeostasis after inflammation. However, different stimulus, some associated with obesity, could sustain inflammation over time, and lead to chronic inflammation. Chronic inflammation would exacerbate the disease and contribute to the onset of other ones.

Obesity-associated hyperleptinemia has been described to stimulate microglial differentiation into M1 phenotype, contributing to neuroinflammation [18]. On the other hand, HDLs have been found reduce the inflammatory status, even in the central nervous system, because HDLs can cross the BBB [25]. Nevertheless, HDLs could lose their anti-inflammatory properties after suffering modifications, such as oxidation and glycoxidation [6].

Even though HDLs and leptin have been tested in microglia and other tissues, their functions have always been analyzed separately. Therefore, it is completely innovative to test them together in the search of new insights of relationship and cooperation between HDLs and leptin in the central nervous system. The purpose of this project was to establish whether HDL and leptin may act as mediators of microglial plasticity and influence neuroinflammation.

The data obtained revealed an existing relationship between leptin levels and HDL oxidation: higher leptin levels positively correlate to HDL oxidation. Therefore, hyperleptinemia is associated with HDL dysfunction; specifically, this result suggests that obesity-associated hyperleptinemia worsens HDL functionality. Obesity itself is described to promote inflammation, and it is associated with insulin and leptin resistance, which contribute to higher blood insulin and leptin levels. Altogether, these things promote an inflammatory storm that would reduce HDLs’ functionality and antioxidant and anti-inflammatory properties, contributing to the rise of pro-inflammatory status.

In this present study, we evaluated the role of healthy and oxidized HDLs in combination with leptin over microglial cells. HDLs derived from normal-weight volunteers showed higher antioxidant capacity compared to HDLs derived from obese volunteers. PON1 activity and quantity was lower in obese-derived HDLs, and this would contribute to less functional and more oxidized HDLs.

HDLs derived from healthy volunteers induced microglial polarization into M2 phenotype, in line with the cytokine expression profile. On the other hand, obese-derived HDLs did not show anti-inflammatory function; even HDLs increased the pro-inflammatory status, because they induced M1 polarization. These results suggest that obese-derived HDLs could act as pro-inflammatory particles, which is in concordance with the low antioxidant capacity that obese-derived HDLs showed.

Additionally, when healthy HDLs were in combination with leptin, HDLs were able to reduce the pro-inflammatory action of leptin, even though it was less efficient than in leptin absence. Moreover, when the HDL concentration was high, it contributed to the increase inflammation. It could imply that a high HDL level in combination with a pro-inflammatory agent, such as leptin, would deplete HDLs from their anti-inflammatory functions and would become a pro-inflammatory agent.

These results can be due to two main different factors: that leptin acts as a pro-inflammatory cytokine in microglia and leads to an oxidative microenvironment that promotes HDL oxidation, or that HDLs in high concentration could easily reverse their functionality and act in combination with leptin to increase inflammation. What is clear is that HDLs vary their functionality according to the environment, and inflammation could reverse HDL’s beneficial properties. Additionally, high HDL levels could worsen the output of an inflammatory agent. Hence, increasing the HDL level when an inflammatory disease is ongoing could not be the best approach to reduce the risk; instead, improving HDL functionality could be a better approach.

HDLs’ relationship with inflammation is clear; however, their role as pro-inflammatory or anti-inflammatory depends on the environment. For future research, HDLs should be tested in combination with other pro-inflammatory agents to clarify the mechanism that changes HDL anti-inflammatory properties in those previously tested as healthy HDLs.

## 5. Conclusions

In conclusion, these results suggest that HDLs play a relevant role in microglial plasticity and neuroinflammation. Specifically, in physiological conditions, HDLs develop an anti-inflammatory function that could be reversed by pro-oxidant and pro-inflammatory microenvironments, such as obesity-associated endotoxemia or hyperleptinemia. Additionally, our results open new opportunities for testing the effect of different pro-inflammatory agents over HDL functionality.

## Figures and Tables

**Table 1:** General characteristics of study population.

Variables	Normal-Weight	Obese	*p*-Value
N	20	20	
Age (years)	31.2 ± 6.7	38.5 ± 4.3	0.060
Smoking (%)	20	30	0.465
Alcohol consumption (%)	45	50	0.751
Physical activity (hours/week)	2.7 ± 2.5	2.4 ± 1.8	0.161
BMI (kg/m2)	23.2 ± 1.5	31.9 ± 4.1	**<0.001**
Waist circumference (cm)	85.3 ± 7.0	124.5 ± 16.1	**<0.001**
Neck circumference (cm)	29.3 ± 0.5	38.4 ± 2.1	**<0.001**
Percent body fat	27.4 ± 0.4	39.6 ± 1.6	**<0.001**
Systolic blood pressure (mmHg)	126.8 ± 5.2	133.8 ± 8.0	0.068
Diastolic blood pressure (mmHg)	82.6 ± 7.1	84.2 ± 6.9	0.902
NEFAs (µmol/L)	387 ± 42	433 ± 45	0.767
Triglycerides (mmol/L)	0.53 ± 0.17	1.29 ± 0.16	0.794
Total Cholesterol (mmol/L)	4.4 ± 0.4	5.2 ± 0.5	0.339
LDL-C (mmol/L)	2.41 ± 0.43	3.03 ± 0.32	0.207
HDL-C (mmol/L)	1.59 ± 0.16	1.11 ± 0.11	0.111
Glucose (mmol/L)	4.45 ± 0.49	5.27 ± 0.62	0.317
HbA1c (%)	5.1 ± 0.4	5.2 ± 0.5	0.339
Insulin (pmol/L)	43.9 ± 4.6	105 ± 18.6	**<0.001**

Data were expressed as % for categorical variables or mean ± SD for normally distributed variables. Values in bold are significant at *p* < 0.05. Abbreviations: BMI, body mass index; NEFAs, non-esterified fatty acids; LDL-C, low-density lipoprotein cholesterol; HDL-C, high-density lipoprotein cholesterol.

## Data Availability

Not applicable.

## Supplementary Materials

The following are available online at https://www.mdpi.com/article/10.3390/biomedicines9111722/s1. Table S1: Detailed information about primers′ sequences used in this study.

## Limitations of the Study

It should be noted that only men were included in the study to isolate HDL.

## References

[1] Ben-Aicha S., Badimon L., Vilahur G. (2020). Advances in HDL: Much More than Lipid Transporters. Int. J. Mol. Sci..

[2] Fotakis P., Kothari V., Thomas D.G., Westerterp M., Molusky M.M., Altin E., Abramowicz S., Wang N., He Y., Heinecke J.W. (2019). Anti-Inflammatory Effects of HDL (High-Density Lipoprotein) in Macrophages Predominate Over Proinflammatory Effects in Atherosclerotic Plaques. Arter. Thromb. Vasc. Biol..

[3] Mahrooz A., Shokri Y., Variji A., Zargari M., Alizadeh A., Mehtarian E. (2021). Improved risk assessment of coronary artery disease by substituting paraoxonase 1 activity for HDL-C: Novel cardiometabolic biomarkers based on HDL functionality. Nutr. Metab. Cardiovasc. Dis..

[4] Sutter I., Velagapudi S., Othman A., Riwanto M., Manz J., Rohrer L., Rentsch K., Hornemann T., Landmesser U., von Eckardstein A. (2015). Plasmalogens of high-density lipoproteins (HDL) are associated with coronary artery disease and anti-apoptotic activity of HDL. Atherosclerosis.

[5] Shih C.-M., Lin F.-Y., Yeh J.-S., Lin Y.-W., Loh S.-H., Tsao N.-W., Nakagami H., Morishita R., Sawamura T., Li C.-Y. (2019). Dysfunctional high density lipoprotein failed to rescue the function of oxidized low density lipoprotein-treated endothelial progenitor cells: A novel index for the prediction of HDL functionality. Transl. Res..

[6] Gao D., Ashraf M.Z., Zhang L., Kar N., Byzova T.V., Podrez E.A. (2020). Cross-linking modifications of HDL apoproteins by oxidized phospholipids: Structural characterization, in vivo detection, and functional implications. J. Biol. Chem..

[7] Soria-Florido M.T., Castañer O., Lassale C., Estruch R., Salas-Salvadó J., Martínez-González M.Á., Corella D., Ros E., Arós F., Elosua R. (2020). Dysfunctional High-density Lipoproteins Are Associated with a Greater Incidence of Acute Coronary Syndrome in a Population at High Cardiovascular Risk: A Nested Case-Control Study. Circulation.

[8] Li B.-Q., Zhong Y.-C., Wang X. (2018). Plasma oxidized high-density lipoprotein and glycated apolipoprotein A-I concentrations in ST-segment elevation myocardial infarction patients with stress hyperglycaemia or high thrombus burden. Upsala J. Med Sci..

[9] Li J., Zhou C., Xu H., Brook R.D., Liu S., Yi T., Wang Y., Feng B., Zhao M., Wang X. (2019). Ambient Air Pollution Is Associated with HDL (High-Density Lipoprotein) Dysfunction in Healthy Adults. Arter. Thromb. Vasc. Biol..

[10] Delbosc S., Diallo D., Dejouvencel T., Lamiral Z., Louedec L., Martin-Ventura J.-L., Rossignol P., Leseche G., Michel J.-B., Meilhac O. (2013). Impaired high-density lipoprotein anti-oxidant capacity in human abdominal aortic aneurysm. Cardiovasc. Res..

[11] Peterson S.J., Shapiro J.I., Thompson E., Singh S., Liu L., Weingarten J.A., O’Hanlon K., Bialczak A., Bhesania S.R., Abraham N.G. (2018). Oxidized HDL, Adipokines, and Endothelial Dysfunction: A Potential Biomarker Profile for Cardiovascular Risk in Women with Obesity. Obesity.

[12] Chen Y., Arnal-Levron M., Hullin-Matsuda F., Knibbe C., Moulin P., Luquain-Costaz C., Delton I. (2018). In vitro oxidized HDL and HDL from type 2 diabetes patients have reduced ability to efflux oxysterols from THP-1 macrophages. Biochimie.

[13] Perakakis N., Farr O.M., Mantzoros C.S. (2021). Leptin in Leanness and Obesity: JACC State-of-the-Art Review. J. Am. Coll. Cardiol..

[14] Zhang L., Reed F., Herzog H. (2020). Leptin signalling on arcuate NPY neurones controls adiposity independent of energy balance or diet composition. J. Neuroendocr..

[15] Tsiotra P.C., Boutati E., Dimitriadis G., Raptis S.A. (2013). High Insulin and Leptin Increase Resistin and Inflammatory Cytokine Production from Human Mononuclear Cells. BioMed Res. Int..

[16] Gerriets V.A., Danzaki K., Kishton R.J., Eisner W., Nichols A.G., Saucillo D.C., Shinohara M.L., MacIver N.J. (2016). Leptin directly promotes T-cell glycolytic metabolism to drive effector T-cell differentiation in a mouse model of autoimmunity. Eur. J. Immunol..

[17] Kiernan K., Maclver N.J. (2021). The Role of the Adipokine Leptin in Immune Cells Function in Health and Disease. Front. Immunol..

[18] Agrawal S., Gollapudi S., Su H., Gupta S. (2011). Leptin activates human B cells to secrete TNF-α, IL-6, and IL-10 via JAK2/STAT3 and p38MAPK/ERK1/2 signaling pathway. J. Clin. Immunol..

[19] Chagas L., Sandre P.C., Ribeiro N.C.A.R.E., Marcondes H., Silva P.O., Savino W., Serfaty C.A. (2020). Environmental Signals on Microglial Function during Brain Development, Neuroplasticity, and Disease. Int. J. Mol. Sci..

[20] Batista C.R.A., Gomes G.F., Candelario-Jalil E., Fiebich B.L., De Oliveira A.C.P. (2019). Lipopolysaccharide-Induced Neuroinflammation as a Bridge to Understand Neurodegeneration. Int. J. Mol. Sci..

[21] Orihuela R., McPherson C.A., Harry G.J. (2016). Microglial M1/M2 polarization and metabolic states. Br. J. Pharmacol..

[22] Plastira I., Bernhart E., Goeritzer M., Reicher H., Kumble V.B., Kogelnik N., Wintersperger A., Hammer A., Schlager S., Jandl K. (2016). 1-Oleyl-lysophosphatidic acid (LPA) promotes polarization of BV-2 and primary murine microglia towards and M1-like phenotype. J. Neuroinflamm..

[23] Ilarregui J.M., Kooij G., Rodríguez E., Van Der Pol S.M.A., Koning N., Kalay H., Van Der Horst J.C., Van Vliet S.J., García-Vallejo J.J., De Vries H.E. (2019). Macrophage galactose-type lectin (MGL) is induced on M2 microglia and participates in the resolution phase of autoimmune neuroinflammation. J. Neuroinflamm..

[24] Liu C., Dai S.K., Shi R.X., He X.C., Wang Y.Y., He B.D., Sun X.W., Du H.Z., Liu C.M., Teng Z.Q. (2021). Transcriptional profiling of microglia in the injured brain reveals distinct molecular features underlying neurodegeneration. Glia.

[25] Sobue A., Komine O., Hara Y., Endo F., Mizoguchi H., Watanabe S., Murayama S., Saito T., Saido T.C., Sahara N. (2021). Microglial gene signature reveals loss of homeostatic microglia associated with neurodegeneration of Alzheimer’s disease. Acta Neuropathol. Commun..

[26] Fung K.Y., Wang C., Nyegaard S., Heit B., Fairn G.D., Lee W.L. (2017). SR-BI Mediated Transcytosis of HDL in Brain Microvascular Endothelial Cells Is Independent of Caveolin, Clathrin, and PDZK1. Front. Physiol..

[27] Johnson J.L., Slentz C.A., Duscha B.D., Samsa G.P., McCartney J.S., Houmard J.A., Kraus W.E. (2004). Gender and racial differences in lipoprotein subclass distributions: The STRRIDE study. Atherosclerosis.

