# Supplementary material for: Obesity-Associated Metabolic Disturbances Reverse the Antioxidant and Anti-Inflammatory Properties of High-Density Lipoproteins in Microglial Cells
Source: Biomedicines. 2021 Nov 19;9(11):1722. doi: 10.3390/biomedicines9111722 (PMC8615358; doi:10.3390/biomedicines9111722)
Supplement: Supplementary file 1 [file biomedicines-09-01722-s001.zip › biomedicines-1462787-Supplementary Materials.pdf]

**Supplementary Table S1.** Sequences of RT-qPCR primers for gene expression analysis.

| Target | No. GenBank | Direction | Sequence (5'→3') |
|---|---|---|---|
| GAPDH | NM_001289726 | Forward<br>Reverse | AACTTTGGCATTGTGGAAGG<br>ACACATTGGGGGTAGGAACA |
| HPRT | NM_013556.2 | Forward<br>Reverse | TATGTCCCCCGTTGACTGAT<br>TGCTCGAGATGTCATGAAGG |
| CCR7 | NM_001301713 | Forward<br>Reverse | CCAGGCACGCAACTTTGAG<br>ACTACCACCACGGCAATGATG |
| iNOS | NM_010927 | Forward<br>Reverse | CACCTTGGAGTTCACCCAGT<br>ACCACTCGTACTTGGGATGC |
| YM1 | NM_009892 | Forward<br>Reverse | ACTTTGATGGCCTCAACCTG<br>AATGATTCCTGCTCCTGTGG |
| ARG1 | NM_007482 | Forward<br>Reverse | GTGAAGAACCCACGGTCTGT<br>CTGGTTGTCAGGGGAGTGTT |
| TNF-α | NM_001278601 | Forward<br>Reverse | AGCCCCCAGTCTGTATCCTT<br>CTCCCTTTGCAGAACTCAGG |
| IL-6 | NM_031168 | Forward<br>Reverse | AGTTGCCTTCTTGGGACTGA<br>TCCACGATTTCCCAGAGAAC |
| IL-10 | NM_010548 | Forward<br>Reverse | CCAAGCCTTATCGGAAATGA<br>TTTTCACAGGGGAGAAATCG |
